# Expressing red fluorescent protein on the surface of *Escherichia coli* using C-terminal domain of autotransporters

**DOI:** 10.22099/mbrc.2024.49860.1956

**Published:** 2025

**Authors:** Khoi-Nguyen Le-Hoang, Thanh-Tan Nguyen, Hieu Tran-Van

**Affiliations:** 1 Labolatory of Biosensors, Faculty of Biology and Biotechnology, University of Science, Ho Chi Minh city, Vietnam; 2 Department of Molecular and Environmental Biotechnology, Faculty of Biology and Biotechnology, University of Science, Ho Chi Minh city, Vietnam; 3 Vietnam National University, Ho Chi Minh city, Vietnam

**Keywords:** Autotransporter; mRFP; C-terminal of EhaA; mRFP-C-terminal complex protein

## Abstract

The Type V secretion system, or “autotransporter”, is a secretion system that enables bacteria to directly export proteins from the cell interior to the extracellular membrane. mCherry is a second-generation monomeric red fluorescent protein that has an improvement in photostability compared to the first generation of RFP. In this research, we conducted the fusion of the mRFP into the C-terminal domain of EhaA – the translocation domain of the autotransporter protein transport system – to investigate the expression of mRFP on the surface of *Escherichia coli,* a model organism commonly utilized in recombinant protein research. The induction of the mRFP-EhaA C-terminal domain complex expression was achieved using isopropyl β-D-1-thiogalactopyranoside (IPTG) and confirmed through SDS-PAGE stained with Coomassie Brilliant Blue and Western blotting using anti-6X His tag antibodies. The surface expression of the mRFP-EhaA C-terminal complex protein was validated through the fluorescent properties of mRFP and further confirmed using fluorescent microscopy. This study laid the groundwork for surface expression on cost-effective Gram-negative bacteria, *E. coli*.

## INTRODUCTION

The expression of proteins on the surface of living cells is noteworthy research, focusing not only on the broader field of biological technology but also on molecular biology specifically. Various surface protein expression systems have been developed in yeast, and for primitive organisms, comprehensive surface expression systems have been established, particularly in Gram-positive bacteria [1, 2]. However, for Gram-negative bacteria, surface expression systems appear less developed or inadequately explored within this microbial group. This is primarily attributed to the complex structure of the outer membrane in Gram-negative bacteria, which poses challenges in developing effective surface expression systems for this bacterial subgroup. Despite the intricate outer membrane structure, Gram-negative bacteria have evolved specialized mechanisms to secrete diverse molecules, ranging from DNA to proteins [3]. These molecules play crucial roles in enabling the bacteria to adapt to various environmental conditions throughout their evolutionary history. Notably, proteins belonging to the Type V secretion system or “autotransporters” exhibit significant potential for developing surface expression systems in Gram-negative bacteria, as they can autonomously secrete through a domain that serves as a translocation channel for polypeptide chains to the outer membrane [4]. Furthermore, both the translocation and functional activity domains reside within the same polypeptide chain. On the other hand, red fluorescent protein (RFP) was first discovered in *Discosoma *sp. in 1999 with a tetramer structure [5]. Monomeric Cherry is the second generation of RFP that has become the most widely used red fluorescence because of its monomeric structure and a low molecular weight that reduces structural interference when fused to other proteins [6]. Therefore, replacing this active domain region with any other peptide sequence can be easily achieved. In this study, we have undertaken the fusion of mCherry into the C-terminal region of EhaA, a translocation domain of the Type Va secretion system responsible for transporting polypeptide chains to the membrane in the autotransporter protein group. This fusion aims to express mCherry and concurrently investigate its surface expression capability using the Type Va secretion system in *Escherichia coli*.

## MATERIALS AND METHODS

**Construction of the expression vector pHEA-mRFP:** *mRFP* gene was amplified from pLEM415-ldhL-mRFP1 plasmid (was a gift from Sujin Bao, Addgene #99842) by PCR with specific primers 941F and 942R, resulting in a 748 bp product. Next, the extracted pHEA plasmid (as a gift from Luis Ángel Fernández, Addgene #168297) was digested with *EcoR*1 and *Cfr*42I (*Sac*II) (Thermo Scientific). The purified *mRFP* gene and the digested plasmid were incubated at a concentration of 1:4. By using the recombinase-free cloning method (RFC), the mixture was incubated at the following temperatures 72°C in 2 minutes, 65°C in 2 minutes, 58°C in 30 minutes, and 10°C in 5 minutes [7]. Then the mixture was transformed into competent *E. coli* DH5α. Finally, the verified pHEA-*mRFP* plasmid was transformed into *E. coli *SHuffle® non-T7 Express cells for protein expression.

**Expression and confirmation of recombinant** **m** **RFP protein in** ***E.coli*** **SHuffle® non-T7 Exp** **r** **ess:** The expression of the recombinant was carried out following the described protocol with certain adjustments [8]. The pHEA-*mRFP* obtained from the previous steps was transformed into *E.* *coli *SHuffle® non-T7 Express, and isopropyl-beta-thiogalactopyranoside (IPTG) was added to a final concentration of 0.5mM. Protein expression was performed at 200 rpm at 30°C for 16 hours, confirmed by SDS-PAGE and Coomassie Brilliant Blue staining, followed by Western Blot probed with HRP-conjugated anti-6X His tag antibody (1:20,000) (ProteinTech).

**Phase separation of membrane proteins by using Triton X-114: **Triton X-114 (TX114) is a non-ionic detergent. It is homogeneous at 0°C, but there is phase separation into detergent (Det) and aqueous (Aq) phases at temperatures above 20°C [9]. During phase separation, hydrophobics are sequestered into the Det phase, while hydrophilic are sequestered into the Aq phase. Therefore, Triton X-114 can be utilized for the concentration hydrophobic proteins attached to the membrane. Triton X-114 will be diluted with 10 mM Tris-HCl (pH 8) to a final concentration of 6% on ice. Next, the induced *E.coli *SHuffle® non-T7 Express cells were prepared by mixing 50µl of 6% Triton X-114 on ice for membrane extraction. The 1.5ml tube was centrifuged for 2 minutes at room temperature and incubate at 37°C for 20 minutes. After incubation, the detergent phase was found as oily droplets at the bottom of the tube, atop the aqueous phase. 

**Confirmation of** **m** **RFP protein in** ***E.coli*** **SHuffle® non-T7 Exp** **r** **ess by using** **f** **luorescence microscopy: **After induction**, ***E.coli *SHuffle® non-T7 Express/pHEA-mRFP cells were harvested by centrifugation at 6,000 rpm for 5 minutes, removing the supernatant, followed by two washes with PBS. For microscopy, 10 μl of the cell suspension was directly mounted onto a microscope slide carrying a thin layer of 0.01 % poly L-lycine in PBS-glycerol with a ratio of 8:2. The ECLIPSE Ti2 inverted microscope (Nikon) equipped with a mercury lamp was used to detect mCherry fluorescence.

**RESULTS** **AND DISCUSSION**

**Figure 1:**
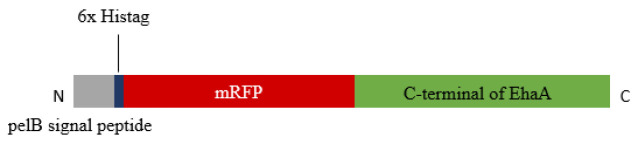
Schematic of designed recombinant mRFP-C-terminal of EhaA protein

The *mRFP* gene was effectively cloned into the expression vector pHEA, resulting in the construction of pHEA-*mRFP* recombinant vector. The inserted sequence verified by Sanger sequencing showed an in-frame cloning as designed in figure 1. 

**Figure 2:**
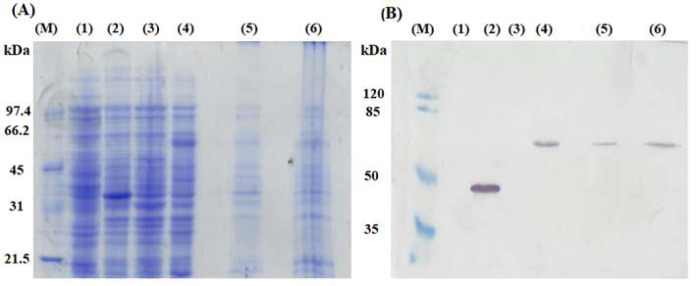
Coomassie Brilliant staining of expressed mRFP-C terminus of the EhaA by SDS-PAGE 10% gel (A) and confirmed by Western blot probed with anti-6X His tag antibody conjugated HRP (B). M, protein marker (A) and pre-stained protein marker (B); 1, *E.coli *SHuffle® non-T7 Express (+IPTG); 2, *E.* *coli *SHuffle® non-T7 Express /pHEA (+IPTG); 3, *E.* *coli *SHuffle® non-T7 Express /pHEA-*mRFP* (-IPTG); 4, *E.coli *SHuffle® non-T7 Express/pHEA-*mRFP* (+IPTG); 5, Aqueous phase; 6, Detergent phase.

The mRFP-C-terminal domain of EhaA complex was induced for expression from *E.coli *SHuffle® non-T7 Express/pHEA-*mRFP* with a molecular mass approximately of about 58 kDa, as confirmed by SDS-PAGE analysis and Western blot. The results shown in figure 2 indicate expression protein bands in lane 4 with the size at about 58 kDa equal to the predicted size. After phase separation, the complex was enriched into the detergent-rich, membrane-bound phase (lane 6) in comparison to the aqueous phase (lane 5). *E.coli *SHuffle® non-T7 Express/pHEA induction also gave rise to the band of 33 kDa (lane 2), indicating that the C-terminal complex of EhaA was expressed. On the other hand, negative controls also did not exhibit any expression bands. 

In addition, the pHEA plasmid had a 6X His tag sequence at the N-terminal, hence, the presence of the mRFP-C-terminal of EhaA complex was confirmed by the anti-6X His tag antibody conjugated HRP in the Western blot. The results showed that the mRFP-C-terminal of EhaA complex excessively expressed in SDS-PAGE gels was recombinant mRFP-C-terminal of EhaA protein complex, and the phase separation indicated that the protein complex was indeed enhanced into the detergent phase. However, the results also concurrently demonstrated the persistence of protein complexes in the aqueous phase, leading us to predict that some complexes remain unexpressed on the membrane of *E.* *coli *SHuffle® non-T7 Express. We posit that the limited spatial availability on the surface of *E. coli* bacteria, which may preclude the expression of certain complexes, lets some protein complexes remain in the cellular milieu. The membrane of *E.coli *SHuffle® non-T7 Express was constructed from a bilayer lipid structure, thus, proteins associated with the membrane inherently possessed hydrophobic properties. Due to its phase separation capability at room temperature, Triton X114 was employed in phase separation experiments to enrich membrane proteins by segregating membrane-bound proteins from the protein mixture [10]. In this process, membrane-associated proteins and membrane-anchored components resided in the hydrophobic phase, while other proteins remained in the aqueous phase. 

**Figure 3:**
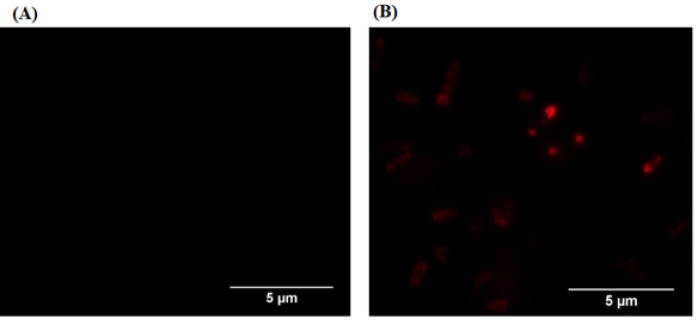
Detection of the mRFP-C-terminal of EhaA complex protein by using fluorescence microscope. A, *E.* *coli *SHuffle® non-T7 Express; B, *E.* *coli *SHuffle® non-T7 Express with expression of the mRFP-C-terminal of EhaA complex.

Furthermore, fluorescence microscopy images in figure 3 also demonstrated signals of mCherry upon excitation at 587 nm and emission detection at 610 nm in *E. coli* transformed with the pHEA-*mRFP* vector compared to *E. coli *SHuffle® non-T7 Express without vector transformation [6]. Therefore, we could conclude that the mRFP-C-terminal complex of EhaA was positively expressed on the surface membrane of *E.* *coli *SHuffle® non-T7 Express.

In this study, we designed, generated, and expressed the mRFP-C-terminal of EhaA protein complex by incorporating the mRFP sequence into the pHEA plasmid. With the capability of fluorescent emission under UV light, mCherry was chosen as a marker to facilitate the confirmation of surface expression of the mRFP-C-terminal of EhaA protein complex through observation under fluorescent microscopy. With this success, pHEA demonstrates its potential for recombinant protein expression on the surface of *E. coli* and its promise in advancing surface display technology. Furthermore, this finding highlights *E. coli *and Gram-negative bacteria as suitable for surface display applications. Therefore, we plan to expand this expression system to display recombinant proteins not only on *E. coli* but also on other Gram-negative bacteria. Particularly, expressing antigens or developing functional foods by displaying enzymes on the surface of probiotic Gram-negative strains, an approach typically explored with Gram-positive bacteria, would be the next objectives in future studies. This shift presents an opportunity to innovate within the field of Gram-negative bacteria [11, 12]. Additionally, alongside therapeutic purposes, expressing other metal-affinity molecules or petroleum hydrocarbon-degrading enzymes on the surface of Gram-negative bacteria to address issues such as oil spill remediation or the treatment of heavy metal-contaminated soil would be an environmentally friendly approach [13, 14].
